# Supplementary material for: Evolution of the Physical and Social Spaces of ‘Village Resettlement Communities’ from the Production of Space Perspective: A Case Study of Qunyi Community in Kunshan
Source: Int J Environ Res Public Health. 2019 Aug 19;16(16):2980. doi: 10.3390/ijerph16162980 (PMC6720272; doi:10.3390/ijerph16162980)
Supplement: Supplementary file 1 [file ijerph-16-02980-s001.pdf]

International Journal of
Environmental Research and Public Health

Supplementary File S1

Questionnaire for the migrant workers in Qunyi Community

Note: your answers to this questionnaire will be kept confidential.

Section 1 The basic information

1. Your age:_________; Gender: ①male ②female; Marriage: ①unmarried ② married

2-1. Your registration status:

①agricultural registered permanent residence ②non-agricultural registered permanent residence.

2-2. Where is your registered residence's address?

①Jiangsu Province ②Other Provinces (Please specify:_______________)

County (City) Name (Please specify):________

3. Your education level:

①primary school and below ②junior high school ③senior high school /vocational high school ④junior college/ undergraduate and above

4-1. Your occupation:

①government employee / public servant ②enterprise worker ③manager / technical staff ④businessman

4-2. Your workplace____________; your income per year_________

5-1. Do you have children?

① Yes (the number of children_____; Age:____) ② No

5-2. If the answer of Questions 5-1 is ①, where do your children live?

① Hometown ② Kunshan ③ elsewhere (Please specify:________________)

Section 2 The conditions of the rented apartment

6-1. The time you came to Kunshan_________; the time you moved to this community_________

6-2. The length of residency in this community___________

6-3. Where did you live before you moved to your present house?

① factory dormitories ②other communities (rent_______yuan per month) ③other (Please specify:_____)

7. Your current renting conditions: renting area___m2; the number of rooms____; the rents___Yuan per month; the ratio of rent to monthly income___%; do you feel the rent is expensive or not?____

8-1. Do your share the apartment with others? Yes________No______

8-2. The total number of people live in your apartment_____

8-3. Who is your roommate?

① Family (spouse) ② Family (including parents and children) ③ Friends ④oneself ⑤ Others (Please specify:______________)

9. Your commuting distance____km^2; Commuting time____minutes; Commuting vehicles:______

10-1. How did you get the information of the apartment?

① advertisement (including posters) ② work unit ③ friends ④Other (Please specify:_________)

10-2. The major determinants of renting the apartment in this community

① low rent ② short commuting distance ③ close to friends and fellow villagers ④ Other (Please specify:_____)

11-1. How do you feel about the environment, security, hygiene, management, etc. of the community?

①satisfied ② dissatisfied ③ Other (Please specify:_____)

11-2. Please specify the main problems of the community:________________________

Section 3 The social interaction and daily activities

12. Your relationship with your landlord:

①General (only pay the rent monthly) ②Good (including frequent contacts and mutual assistance) ③Other (Please specify:_____)

13. How did you get the information of your present job?

①by yourself ②fellow villagers ③the landlord ④locals (including neighbors, landlords, etc.) ⑤friends made after came to Kunshan ⑥employment agency ⑦Other (Please specify:________)

14. Who do you usually associate with?

①relatives ②fellow villagers ③co-workers ④locals (including neighbors, landlords, etc.) ⑤friends made after came to Kunshan ⑥Other (Please specify:________)

15. Your views on the other three types of residents (landless farmers, local New Kunshan citizens, and alien New Kunshan citizens) (Please specify):____

____

16. If you encounter problems in housing, employment, family affair, economic issues, children's education, etc., who do you usually consult with?

①family ②relatives ③fellow villagers ④co-workers ⑤locals (including neighbors, landlords, etc.) ⑥ friends made after came to Kunshan ⑦Other (Please specify:________)

17. Your daily activities after work and the location：(Please specify):____________________________________

____

Section 4 The envision of work and life

18. If you have a chance, would you like to move to a better community?

① really hope ②hope ③not interested ④ have no idea

19. How many years do you plan to move to a better community?

①Within 1 year ②1-2 years ③2-3 years ④3-5 years ⑤Live here for a long time ⑥No idea

20-1. If you can move to a better community, where would you like to choose?

①hometown ②the Kunshan City ③near your work ④the nearby Cities

20-2. Please write down the reasons for your choice:___________________

21. Please introduce your work envisions:

①do farming in hometown ②do non-agricultural work in hometown (Please specify:________) ③continue to work in factories in Kunshan or elsewhere ④find another job in Kunshan or elsewhere (Please specify:________) ⑤Other (Please specify:________)

22. Do you want to get a local non-agricultural residence registration in Kunshan and become a new Kunshan citizen:

①really hope ②hope ③not interested ④ have no idea

Thank you for your cooperation!

© 2019 by the authors. Submitted for possible open access publication under the terms and conditions of the Creative Commons Attribution (CC BY) license (http://creativecommons.org/licenses/by/4.0/).
